# A pragmatic cluster randomized controlled trial of early intervention for chronic obstructive pulmonary disease by practice nurse-general practitioner teams: Study Protocol

**DOI:** 10.1186/1748-5908-7-83

**Published:** 2012-09-07

**Authors:** Helen K Reddel, Sarah M Dennis, Sandy Middleton, CP Van Schayck, Alan J Crockett, Iqbal Hasan, Oshana Hermiz, Sanjyot Vagholkar, Guy B Marks, Nicholas A Zwar

**Affiliations:** 1 School of Public Health and Community Medicine, University of New South Wales, Sydney, NSW, 2052, Australia; 2 Woolcock Institute of Medical Institute, University of Sydney, Sydney, NSW, Australia; 3 Centre for Primary Health Care and Equity, University of New South Wales, Sydney, NSW, 2052, Australia; 4 National Centre for Clinical Outcomes Research, Australia, Australian Catholic University, Sydney, NSW, Australia; 5 Research School Caphri, Maastricht University, Maastricht, The Netherlands; 6 Discipline of General Practice, School of Population Health And Clinical Practice, The University of Adelaide, Adelaide, 5005, South Australia; 7 General Practice Unit, Fairfield Hospital, South Western Sydney Local Health District/School of Public Health & Community Medicine, University of New South Wales, Sydney, NSW, 2052, Australia; 8 Woolcock Institute of Medical Research, University of Sydney and Department of Respiratory Medicine, Liverpool Hospital, Liverpool, NSW, 2170, Australia

## Abstract

**Background:**

Chronic Obstructive Pulmonary Disease (COPD) is a leading cause of disability, hospitalization, and premature mortality. General practice is well placed to diagnose and manage COPD, but there is a significant gap between evidence and current practice, with a low level of awareness and implementation of clinical practice guidelines. Under-diagnosis of COPD is a world-wide problem, limiting the benefit that could potentially be achieved through early intervention strategies such as smoking cessation, dietary advice, and exercise. General practice is moving towards more structured chronic disease management, and the increasing involvement of practice nurses in delivering chronic care.

**Design:**

A pragmatic cluster randomised trial will test the hypothesis that intervention by a practice nurse-general practitioner (GP) team leads to improved health-related quality of life and greater adherence with clinical practice guidelines for patients with newly-diagnosed COPD, compared with usual care. Forty general practices in greater metropolitan Sydney Australia will be recruited to identify patients at risk of COPD and invite them to attend a case finding appointment. Practices will be randomised to deliver either practice nurse-GP partnership care, or usual care, to patients newly-diagnosed with COPD.

The active intervention will involve the practice nurse and GP working in partnership with the patient in developing and implementing a care plan involving (as appropriate), smoking cessation, immunisation, pulmonary rehabilitation, medication review, assessment and correction of inhaler technique, nutritional advice, management of psycho-social issues, patient education, and management of co-morbidities.

The primary outcome measure is health-related quality of life, assessed with the St George’s Respiratory Questionnaire 12 months after diagnosis. Secondary outcome measures include validated disease-specific and general health related quality of life measures, smoking and immunisation status, medications, inhaler technique, and lung function. Outcomes will be assessed by project officers blinded to patients’ randomization groups.

**Discussion:**

This study will use proven case-finding methods to identify patients with undiagnosed COPD in general practice, where improved care has the potential for substantial benefit in health and healthcare utilization. The study provides the capacity to trial a new model of team-based assessment and management of newly diagnosed COPD in Australian primary care.

**Trial registration:**

ACTRN12610000592044\

## Background

Chronic obstructive pulmonary disease (COPD) is a leading cause of morbidity and mortality [1], ranked globally in 2002 as the fifth leading cause of death [2] and the seventh leading cause of disease burden after ischaemic heart disease and stroke. In Australia, COPD is the fourth leading cause of death for males (4.2% of all deaths) and the sixth leading cause of death for women (3.3% of all deaths); it is also a contributory cause of death in many patients with coronary heart disease or cancer [3]. In the Burden Of Obstructive Lung Disease survey (BOLD), the prevalence of airflow limitation (GOLD Stage II or higher) in Australians aged ≥40 years was 10.8% [4], whereas the prevalence of doctor-diagnosed COPD in the same population was 5.9% [5].

Clinically, COPD is characterised by airflow limitation that is not fully reversible, and is associated with an enhanced chronic inflammatory response to noxious particles or gases [6]. Patients typically present with breathlessness, cough, and sputum production. The most important cause in developed countries is cigarette smoking, and up to 50% of smokers may eventually develop clinically significant COPD [7].

Even mild to moderate COPD is associated with impaired health status [8]. Patients with COPD have increased healthcare utilisation before diagnosis, raising the possibility that earlier diagnosis may allow more rational and directed use of healthcare resources [9]. The feasibility of a COPD case-finding approach has been established. For example, in an Australian study in 2007, 20% of patients identified as being at risk of COPD responded to invitations to be screened and of these 20% had a new diagnosis of COPD on spirometric criteria [10]. This is similar to findings from primary care studies in other countries [11-13]. For diagnosis of COPD, spirometry is required [6], and standards have been developed for the performance of spirometry in primary care [14]. Practice nurses can feasibly and successfully undertake spirometry [13,15]; as for other health professionals, approximately six hours of training are required [16].

Clinical practice guidelines have been developed and disseminated for the diagnosis and management of COPD [6], including in Australia (COPD-X guidelines) [17]. Despite the high level evidence for the efficacy of guidelines-based interventions, the care provided for patients with COPD in community settings indicates low levels of awareness and implementation of these guidelines [18]. Medication use is often not in accordance with guidelines [19,20], and a high proportion of patients prescribed inhalers use them incorrectly [21,22].

Effective treatment for COPD improves symptoms, prognosis, and quality of life. Smoking cessation is the most effective measure to reduce progression of the disease [23]. Because smoking cessation may become less effective at altering the course of disease in patients with severe COPD, interventions that target patients with mild and moderate disease may be more effective [24]. In the Lung Health Study [25] smokers with early COPD who were assigned to a smoking cessation intervention had fewer respiratory symptoms after five years follow-up than those who were not. Smokers diagnosed with COPD [26] or who are told their ‘lung age’ [27] may be more likely to cease smoking.

General practice is well placed to diagnose COPD and provide early intervention and longer-term management [28,29]. Care planning and a team approach are effective in the management of chronic disease. Care planning by general practitioners (GPs) has been shown to improve the clinical outcomes for other chronic diseases such as diabetes [30] and asthma [31], but GPs need more support to develop and implement multidisciplinary care plans [32,33] Current guidelines recommend the use of multidisciplinary care plans in the management of patients with COPD [17].

Practice nurses are increasingly contributing to chronic disease management. Specialised nurses have contributed to the care of patients with diabetes and COPD, and there is evidence of improvements in patient self-care, quality of life, and satisfaction [34]. A Cochrane review [35] of nursing outreach programs for COPD found significant gains in health-related quality of life for patients with moderate COPD, but the review highlighted the lack of high quality studies and concluded that further study was required.

While the role of specialist nurses in contributing to the care of patients with COPD has been examined [35-37] and some evidence of benefit in disease-specific quality of life has been found, there are few studies examining the potential role of practice nurses working in partnership with GPs in providing more coordinated, integrated, and evidence-based care for patients with newly-diagnosed COPD.

### Study aims

The primary aim is to assess the effectiveness of early intervention by a GP-nurse team applying evidence-based guidelines, compared with usual care, in the assessment and management of patients newly diagnosed with COPD. Secondary aims are to assess the acceptability of the two management approaches to GPs, nurses and patients, and to assess the utility of the COPD Diagnostic Questionnaire (CDQ) and COPD Assessment Test (CAT) in an Australian population.

### Study hypothesis

The study hypothesizes that intervention by a GP-practice nurse team leads to improved health-related quality of life and greater adherence with clinical practice guidelines for patients with newly-diagnosed COPD, compared with usual care.

## Methods

### Study approvals

The PELICAN study (Primary care EarLy Intervention for Copd mANagement) is a pragmatic cluster randomized controlled study with two treatment arms, which is being conducted in greater metropolitan Sydney, Australia. The study is funded by the National Health and Medical Research Council of Australia (Project Grant No. 630421). Ethical approval has been obtained from the Human Research Ethics Committee, University of New South Wales (HREC 10015). The project has been registered with the Australian Clinical Trials Registry (ACTRN12610000592044).

### Study design

This pragmatic cluster randomized controlled trial will test the hypothesis that early intervention by a practice nurse-GP partnership will improve outcomes for patients newly diagnosed with COPD, compared with usual care. Because the intervention involves a team approach by treating practitioners, randomization will occur at the practice level rather than at the level of individual patients (CONSORT guidelines for cluster trials [38,39]). All participating practices will use case-finding methods to identify a cohort of patients with newly diagnosed COPD.

**Figure 1:**
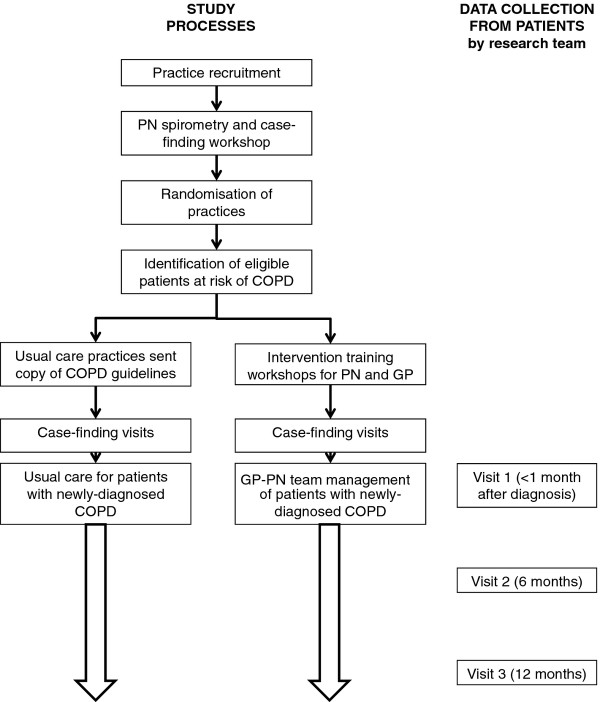
Study and data collection processes.

The research plan, identifying recruitment strategies, randomization, data collection points, and the relationships between intervention and control groups in the two arms are summarised in Figure 1. Because it is a complex intervention with potential for variable implementation, there will be a focus on process evaluation as well as quantitative outcome measures.

### Inclusion criteria

#### GPs and practice nurses

Practices will be eligible to participate if they have computer-based patient records, employ at least one practice nurse, and have a spirometer. More than one GP per practice may be involved in the study. Written informed consent will be obtained from GP(s) and practice nurse(s).

#### Patients

Patients will be eligible for inclusion if they have attended the practice at least twice, with at least one visit in the preceding 12 months, and have risk factors for COPD (aged 40 to 85, and with a documented history of smoking). Patients will be excluded if they have a recorded diagnosis of COPD, are unable to understand English sufficiently to complete study questionnaires or procedures, or have cognitive impairment.

### Randomization and allocation concealment

Practices will be randomized to deliver either a practice nurse-GP team management approach, or usual care, to patients with newly-diagnosed COPD. Randomization will be undertaken after the nurses have completed spirometry and case-finding training, and before case-management training for the intervention group. Randomization will be carried out independently of study and practice staff by a statistician separate to the study team using a computer-generated randomization program, with a minimisation algorithm to ensure a balance of practice characteristics that could potentially affect study outcomes. These characteristics are: practice size, as indicated by the number of practice nurses (1 or >1); socio-economic status, classified according to Socio-Economic Indexes for Areas (SEIFA, [40]) (low: SEIFA 1-5, high: SEIFA 6-10); and participation by the GP in significant (>6 hours) COPD education activities within the last six months (yes or no). Allocation concealment will be ensured as group allocation will be conducted at the same time as randomization. Practices will be informed about their group allocation by fax.

### Blinding

Participating GPs, practice nurses and patients will not be blinded to the true aims of the study, nor to their randomization group. Project officers, who will collect study outcome measures, will be blinded to group allocation as will the statistician undertaking the analyses.

### Recruitment

#### GP and practice nurse recruitment

Recruitment will be conducted with assistance from Divisions of General Practice (local organisations of GPs) in the greater Sydney area. GPs and practice nurses will be provided with an information sheet and asked to provide written consent. All practices expressing an interest in taking part in the project will be visited by an investigator (SD) to discuss the study, answer questions, and obtain consent from the GP and practice nurse. Further recruitment strategies will include provision of study information packs at GP and practice nurse workshops and conferences, and by email to members of the Australian Practice Nurses Association.

#### Intervention and Control Groups: Patient recruitment

For both randomization groups, patient recruitment will involve three stages:

1. Potentially eligible patients, i.e., those at risk of COPD (aged 40 to 85, with documented smoking history) will be identified through a search of the practice electronic records by research assistants and practice staff who have no involvement in patient care.

2. Eligible patients will be recruited by a letter from the practice inviting them to a case-finding visit and management of COPD, if diagnosed. The letter will include a brief description of the research and an invitation to participate, and a response form and reply-paid envelope. Non-responders will be followed up by GP practice staff by telephone.

3. Patients who contact the practice in response to the letter will be given an appointment for the case-finding visit with the practice nurse. The consent form will be signed at the start of the case-finding visit.

For eligible patients who decline to participate in a case-finding visit, basic de-identified information (age, gender, language spoken) will be collected from the practice to allow examination of response bias.

### Training of GPs and practice nurses

#### Intervention and Control Groups—case-finding training of practice nurses for diagnosis of COPD

All practice nurses, before randomization, will receive training in case-finding for diagnosis of COPD. For practice nurses, this comprises a full-day workshop covering the case-finding approach to diagnosis of COPD, and an evening follow-up workshop approximately two weeks later. Because spirometry is essential to the accurate diagnosis and assessment of COPD [6], detailed training in the performance and interpretation of spirometry will be provided (total, eight hours), based on the American Thoracic Society and the European Respiratory Society (ATS/ERS) lung function guidelines of 2005 [41,42]. Nurses will receive practical training on their practice’s own spirometer, and calibration of each spirometer will be checked during the workshop. The follow-up workshop will include revision of spirometry and the processes for the case-finding appointment.

#### Intervention Group only—training in GP-nurse team management of newly-diagnosed COPD

In practices randomized to the GP-practice nurse partnership management arm, the nurses and GPs will attend further workshops to receive practical training in team-based management of COPD. These additional workshops for GPs and practice nurses in the GP-practice nurse partnership intervention will be provided by the study team including a nurse academic, a GP academic, and a respiratory physician. The intervention workshops will comprise a full day for practice nurses, a distance learning activity for GPs, and an evening workshop for both practice nurses and GPs.

The one-day training workshop for nurses will cover: pathophysiology and assessment of COPD; smoking cessation including use of lung age as a motivational tool [27]; COPD management according to Australian guidelines [17]; prescribing guidelines; assessment of patients’ inhaler technique and education to improve technique [43]; the role of pulmonary rehabilitation; the management of exacerbations; the use and value of nurse/GP joint care planning; information about reimbursement codes for relevant activities; and education about self-efficacy theory, motivation, change management, teamwork, and fostering partnerships. The program will be based on that used in an earlier study [44], with adaptation to address specific issues for patients with newly-diagnosed COPD. It will foster a culture of positive organisational change through team building and promotion of collaborative practice [45]. Practice nurses will be provided with a folder containing workshop notes and presentations.

GPs will complete a computer-based distance learning activity and quiz on the management of COPD according to the COPDX guidelines. The evening three-hour workshop for GPs and practice nurses will focus on the importance for each GP-practice nurse team to clarify individual elements of their roles, because lack of understanding has been shown to be a barrier to collaborative practice [46,47]. Each practice will be provided with a copy of current Australian COPD guidelines [17].

#### Control group training

After case-finding training, practices that are randomized to usual care will be provided with a print copy of the Australian COPD guidelines [17]. At the end of the study, after conclusion of all data collection for all practices, GPs and practice nurses in the control group will be offered a workshop on assisting patients to optimise their inhaler technique.

### Study procedures

#### Intervention and Control Groups—case-finding appointments

At the case-finding visit, the practice nurse will first obtain written informed consent for the case-finding process and for participation in the study if COPD is diagnosed; patients will also be asked to consent to release of their contact details to the project officer for arranging data collection visits. The practice nurse will administer a questionnaire including demographic data and the COPD Diagnosis Questionnaire (CDQ, [48]), without calculation of the CDQ score, and will perform spirometry before and after two inhalations of salbutamol. Practice nurses will be provided with a computer-based toolkit [49] to assist in diagnosis of COPD, which will be based on post-bronchodilator FEV1/FVC <0.7 [6,17]. The patient consent and demographic/contact details, spirometry printout, diagnosis, and CDQ responses will be faxed to the project officer. Quality of all spirometry traces will be reviewed by one investigator (AC), and if necessary, feedback or additional training in spirometry will be provided to the practice nurse, initially by telephone, with face-to-face support, if needed. Patients will be included in the study on the basis of the COPD diagnosis assigned by the nurse and/or GP from the case-finding spirometry.

All current smokers—irrespective of the results of spirometry or practice randomisation—will be offered smoking cessation advice and resources at the case-finding visit. Patients with newly diagnosed COPD will be offered management and follow-up, as below, according to whether the practice is in the intervention or control group. If spirometry appears abnormal for other reasons (bronchodilator reversibility suggesting asthma, or restrictive spirometry), the patient will be referred to the GP.

#### Intervention group—delivery of GP-Practice nurse team management for newly-diagnosed COPD

In the intervention practices, the practice nurse will work in partnership with the GP and patient to develop a care plan for patients newly-diagnosed as having COPD. The workbooks provided during the intervention group training workshops will prompt the nurse and GP to include relevant components of the following in the care plan:

1. Smoking cessation: advice based on Smoking Cessation Guidelines for Australian General Practice [50].

2. Immunization: influenza and pneumococcal vaccination status will be assessed relative to local guidelines [51], and immunization recommended and provided where appropriate.

3. Exercise: recommendation about regular exercise for all patients regardless of COPD severity; patients with moderate or severe COPD provided with details of their local pulmonary rehabilitation provider.

4. Medication review: review of current medications and education about potential future indications for pharmacotherapy. For patients already using any inhaler, inhaler technique will be checked using published checklists [43]. Patients for whom inhaled medication is indicated will be educated on the appropriate use of these using established protocols [43].

5. Nutrition: Advice about diet and exercise and referral to a dietician if appropriate for overweight or underweight patients.

6. Psychosocial issues: identification and management of anxiety and/or depression.

7. Co-morbidities and complications of COPD will be assessed and managed.

8. Patient education: patients will be provided with written information about COPD (from Australian Lung Foundation http://www.lungfoundation.com.au/) and its management and with information about local patient support groups which provide emotional support and self-management information.

The nurse and GP will work in partnership with the patient to implement care plans using appropriate prescribing, investigations, referral, and follow-up consultations with the GP, and visits or telephone consultations with the practice nurse. Patients will be added to a COPD Register within the practice to facilitate appropriate recall and reminder notifications and monitor patient progress. This will allow recording of implementation milestones in patient care plans, outstanding actions, and review arrangements. The guideline-based care plan for each patient will seek to optimise management, improve function, prevent deterioration, and enhance patient knowledge and skills. It may be informal, or may be documented in line with government-funded reimbursement programs for chronic disease care [32].

#### Control group: usual care for newly-diagnosed COPD

In the control practices, at the completion of the case-finding visit, the nurse will recommend that patients with newly-diagnosed COPD should see their GP for management of the condition. Usual care for the purpose of this study will thus be the normal pattern of care of COPD by the GP; to ensure that all GPs have access to current Australian COPD guidelines, they will be provided with a print copy [17]. All current smokers will be offered brief smoking cessation advice at the case-finding visit, regardless of the results of spirometry.

#### Intervention and control groups: mentoring of practice nurses

Brief periodic mentoring will be provided to all practice nurses by experienced registered nurses in order to facilitate compliance with study processes, to enhance recruitment and retention of patients, and to provide a standardised framework for responding to questions. Intervention and control practices will receive similar levels of spirometry mentoring. Intervention practices alone will receive mentoring relating to GP-nurse case management of COPD. Mentoring of intervention and control practices will be carried out by two different registered nurses to avoid any cross-contamination between randomisation groups.

### Outcome measures

#### Data collection—patients

Once the project officer has received the consent form, contact details, and spirometry worksheet for a patient, they will arrange a visit as soon as possible for baseline assessment, either at the patient’s home or at the GP’s practice according to patient preference. The project officer will collect demographic data including age, gender, body mass index, employment status, education, and country of birth, and data for outcome and process measures. Further data collection will occur at 6 months (by telephone and mail) and 12 months (face to face).

#### Outcome measures—patients

The primary outcome measure will be disease-specific quality of life assessed by the St George’s Respiratory Questionnaire (SGRQ [52]). Secondary outcome measures include: patient awareness of COPD diagnosis, COPD Assessment Test (CAT, a disease specific QOL measure [53]); smoking status by self-report and from carbon monoxide analysis (Smokerlyzer, Bedfont Scientific Ltd, Maidstone, UK) and readiness to quit if applicable; immunisation rates; medication including appropriate inhaler prescription and effective inhaler use; referral for pulmonary rehabilitation or referral for or provision of exercise prescriptions; disease related knowledge [54]; patient satisfaction; healthcare utilisation; and lung function. The relationship between objectives, outcomes, measures, and hypothesis is outlined in Table 1.

**Table 1:** Relationships between objectives, outcomes, measures and hypotheses

**Objective**	**Hypothesis**	**Outcome**	**Measure**	**Visit**
To assess the effectiveness of early intervention by a GP-practice nurse partnership, in patients newly identified as having COPD	Early intervention by a GP–practice nurse partnership will improve outcomes for patients with COPD, compared with usual care	Improvement in disease related quality of life	St George’s Respiratory Questionnaire (SGRQ) [52]	1,2,3
			COPD Assessment Test (CAT)	1,2,3
		Improved smoking cessation rates	Participation in smoking cessation program; quit rates (self-report)	1,2,3
			Validation of smoking cessation (where applicable) by carbon monoxide analysis	1,3
		Improved compliance with COPD immunisation recommendations	Influenza and pneumococcal vaccination status (self-reported)	1,3
		Improved prescribing and use of medications for COPD	Audit of prescribing against COPD-X guidelines	1,2,3
			Inhaler technique score using published checklists [43]	1,3
		Increased referral for pulmonary rehabilitation	Completion of pulmonary rehabilitation (self-reported)	1,2,3
		Increase in disease related knowledge	COPD knowledge questionnaire score [54]	1,3
		Improved general health status	General health status question (using preliminary question from SGRQ) [52].	1,2,3
		Slower decline in lung function	Post-bronchodilator FEV_1_	1,3
To assess the acceptability and feasibility of team-based vs conventional management of patients with COPD		Improved team-work between GP and practice nurse	Collaborative Practice Scale, VAS scale for team management of COPD	End of study
		Barriers and facilitators to team-work management of COPD	Process measures relating to GP and PN visits.	1, 2, 3
			Semi-structured interviews with GPs, PNs, patients	End of study

#### Process measures

At each visit/telephone call, patients will be asked the number of times they have visited the GP and/or practice nurse (total, and COPD-related), and the COPD-related areas addressed. At the end of the study, GPs and practice nurses in both groups will be asked to rate the extent to which they practice team-based management of COPD, on a visual analogue scale. Practice nurses and GPs will complete the Collaborative Practice Scale [55] to assess the ‘interactions between nurses and GPS that enable the knowledge and skills of both professionals to synergistically influence patient care’ [55].

Semi-structured interviews at the conclusion of the project with the practice nurses, GPs, and patients will examine satisfaction with the program and will explore the effects and value of the nurse input into COPD diagnosis and care, and barriers and facilitators to the team-management approach. The software package NVivo® will be used to facilitate coding and exploration of the data.

#### Reimbursement

The project will provide the following support to participating practices:

1. Intervention and control practices: Reimbursement for the practice nurse attendance at the COPD diagnosis and spirometry workshop (AUD270); payment for the practice nurse to undertake the medical record search (AUD500); reimbursement for practice nurse time for case-finding consultations (AUD38.50 for each appointment, for up to 60 patients per practice); continuing professional development points for workshop attendance.

2. Intervention group: reimbursement for practice nurse attendance at the COPD team management workshop (AUD270); reimbursement for GP attendance at the team management workshop (AUD450); category two continuing professional development points for GPs.

Once a diagnosis of COPD is made, any medical or practice nurse consultations that are required for clinical management may be funded through normal practice processes.

Small gifts (food baskets) will be provided to practice reception staff as thanks for their assistance in making follow-up telephone calls.

#### Data analysis

Data collation will be managed using Access, and data analysis undertaken using the Statistical Package for the Social Sciences (SPSS) by a statistician blind to group allocation. Analysis will be by intention to treat, i.e., by the diagnosis assigned by the practice nurse/GP on the basis of case-finding spirometry, including patients whose diagnosis of COPD is not confirmed by the project officer. Intra-cluster (practice) correlation coefficients will be determined and published for all primary outcome variables to assist future research.

The effect of the intervention on outcomes measured on a continuous scale (such as SGRQ score) will be estimated and tested using mixed model analysis of variance in which time and treatment group will be fixed effects and GP practice and subject nested within practice will be random effects. The effect of the intervention on the dichotomous variables (such as smoking and vaccination status) will be analysed using generalised estimating equations with a logistic link and a model structure that is analogous to that described above.

#### Sample size calculations

The sample size calculation is based on a minimum clinically important difference for the primary outcome measure (SGRQ) of 4.0 [52], between subject standard deviation in SGRQ of 13 in a similar population [54], intra-cluster correlation coefficient of 0.01, and a resultant design effect of 1.09 for a cluster size of 10. With this design effect, a sample size of 200 patients completing each arm will provide >80% power to detect a difference of 4 or greater in SGRQ (calculations in PASS software). Forty practices will each invite 300 patients to attend a case-finding appointment, and within each practice this is expected to produce 12 patients with a new diagnosis of COPD; loss to follow-up of 20% by 12 months has been assumed. The projected recruitment rate is derived from the response rate to invitation (25%), and the yield of new diagnoses (21%) in our recently completed case-finding project [10].

This sample size will also confer 90% power to identify a doubling of the expected rate of correct inhaler use (from 20% to 40%) at a significance level of 5% (based on an ICCC of 0.1, and design effect of 2.1).

## Discussion

There is substantial evidence that COPD is under-diagnosed in the community, and that even once a diagnosis of COPD is made, many patients are not managed according to evidence-based guidelines. By the time the diagnosis is made, COPD is often far-advanced, limiting the benefit that could potentially be achieved through early intervention with strategies such as smoking cessation, dietary advice, and increased exercise. This study will use proven case-finding methods in an at-risk population to identify new patients with COPD, followed by a pragmatic cluster randomised study design to test a novel intervention designed to improve the implementation of evidence-based guidelines for newly-diagnosed COPD. Practice nurses and GPs will be trained in practical strategies for assessing and managing COPD, and in working as a team in managing patients with chronic diseases. Elements of the intervention have been piloted and found to be feasible. The impact of this team-based approach to COPD management on quality of life will be compared with usual care using a widely used and validated tool (SGRQ). Other outcome measures will evaluate the concordance of patient management with evidence-based guidelines for management of COPD, using instruments with demonstrated validity and reliability, which are congruent with the recommendations of the ERS/ATS Task Force on COPD trials [56]. In addition, the acceptability of the team management approach to GPs and practice nurses, and barriers and facilitators to its implementation, will be evaluated with structured interviews at study end.

This study targets COPD patients in general practice where the contact with potential patients with mild and moderate COPD is greatest, and where improved care has the potential for substantial health benefit. Because COPD is a major contributor to the burden of disease in countries such as Australia, improved care is of great public health significance. If successful, the trial will provide a model for care of COPD patients in the community that is feasible and sustainable. Publication of the results of the trial will influence policy and practice on how care for COPD is provided at a national and international level.

### Sustainability of the intervention

In this pragmatic study, the intervention deliberately makes use of and augments existing practice structures, staffing, and approaches to chronic disease management. While there is potential for nurses to undertake advanced practice in primary care, this study will not require practice nurses to work at an advanced practice level. For this study, the role of the practice nurse is focussed on patient assessment; counselling; education; and liaison with medical and allied health colleagues, using expertise that forms part of the skills base of registered nurses. The intervention is thus potentially generalizable and easily transferable.

## Abbreviations

BOLD, Burden of Obstructive Lung Disease; CAT, COPD Assessment Test; CDQ, COPD diagnostic questionnaire; COPD, Chronic obstructive pulmonary disease; FEV_1_, Forced expiratory volume in one second; GOLD, Global Initiative for Chronic Obstructive Pulmonary Disease; GP, General practitioner; PELICAN, Primary care EarLy Intervention for Copd mANagement; PN, Practice nurse; SGRQ, St George’s Respiratory Questionnaire.

## Competing interests

HR has participated on COPD advisory committees for Novartis, has spoken about COPD guidelines at symposia funded by AstraZeneca and Boehringer Ingelheim, has received travel support from AstraZeneca, GlaxoSmithKline and Novartis, and has received independent research funding from GlaxoSmithKline for an investigator-initiated COPD study. GBM is on an advisory board for Novartis and his institution has received funds from AstraZeneca for consultancies. He has spoken at education symposia sponsored by AstraZeneca and GlaxoSmithKline. NZ has provided expert advice on smoking cessation education programs to Pfizer Pty Ltd and GlaxoSmithKline Australia Pty Ltd and has received support to attend smoking cessation conferences. Other authors have no competing interests.

## Authors’ contributions

The study was conceived by JB, NZ and GM, and all authors contributed to the study design. CPvS advised on the design of the CDQ utility study. AC designed the spirometry toolkit for diagnosis of COPD. HR wrote the initial draft of the manuscript. All authors contributed to and approved the final version of the manuscript.
